# Stress and Distress during the COVID-19 Pandemic: The Role of Neighborhood Context

**DOI:** 10.3390/ijerph19052779

**Published:** 2022-02-27

**Authors:** Michelle C. Kondo, Erica Felker-Kantor, Kimberly Wu, Jeanette Gustat, Christopher N. Morrison, Lisa Richardson, Charles C. Branas, Katherine P. Theall

**Affiliations:** 1 Northern Research Station, USDA Forest Service, 100 N. 20th St, Suite 205, Philadelphia, PA 19103, USA; 2 Department of Social, Behavioral, and Population Sciences, Tulane University School of Public Health and Tropical Medicine, New Orleans, LA 70112, USA; efelkerk@tulane.edu (E.F.-K.); kwu6@tulane.edu (K.W.); 3 Department of Epidemiology, Tulane University School of Public Health and Tropical Medicine, New Orleans, LA 70112, USA; gustat@tulane.edu (J.G.); ktheall@tulane.edu (K.P.T.); 4 Department of Epidemiology, Mailman School of Public Health, Columbia University, New York, NY 10032, USA; cm3820@cumc.columbia.edu (C.N.M.); ccb2166@cumc.columbia.edu (C.C.B.); 5 Department of Epidemiology and Preventive Medicine, School of Public Health and Preventive Medicine, Monash University, Melbourne, VIC 3004, Australia; 6 Institute of Women and Ethnic Studies, Research and Technology Foundation, Inc., 2021 Lakeshore Drive, Suite 220, New Orleans, LA 70112, USA; lrich@iwesnola.org

**Keywords:** psychological distress, neighborhood characteristics, parks, greenspace, crime, walkability

## Abstract

Neighborhoods play a central role in health and mental health, particularly during disasters and crises such as the COVID-19 pandemic. We examined changes in psychological distress following the pandemic, and the potential role of neighborhood conditions among 244 residents of New Orleans, Louisiana. Using modified linear regression models, we assessed associations between neighborhood characteristics and change in psychological distress from before to during the pandemic, testing effect modification by sex and social support. While higher density of offsite alcohol outlets (β = 0.89; 95% CI: 0.52, 1.23), assault rate (β = 0.14; 95% CI: 0.03, 0.24), and walkable streets (β = 0.05; 95% CI: 0.02, 0.07) in neighborhoods were associated with an increase in distress, access to neighborhood parks (β = −0.03; 95% CI: −0.05, −0.01), collective efficacy (β = −0.23; 95% CI: −0.35, −0.09), and homicide rate (β = −1.2; 95% CI: −1.8, −0.6) were associated with reduced distress related to the pandemic. These relationships were modified by sex and social support. Findings revealed the important but complicated relationship between psychological distress and neighborhood characteristics. While a deeper understanding of the neighborhoods’ role in distress is needed, interventions that target neighborhood environments to ameliorate or prevent the residents’ distress may be important not only during crisis situations.

## 1. Introduction

The Coronavirus disease 2019 (COVID-19) pandemic has had considerable negative effects attributable to direct traumatic experiences associated with illness from the disease as well as indirect consequences of quarantine measures, employment loss, and education restriction. Research documents decreased psychological well-being and increased anxiety and depression compared to pre-pandemic prevalence [1]. A comparison of findings from the National Health Interview Survey pre-COVID-19 and responses during the pandemic found that U.S. adults were eight times more likely to suffer symptoms of serious psychological distress (an emotional state that can look like anxiety or depression [2]), and three times more likely to suffer moderate psychological distress [3]. Stress is the body’s physiologic response to real or perceived external threats, which can be an important protective mechanism (McEwen, 1998). However, stress experienced chronically and coping mechanisms (McEwen and Gianaros, 2010) can lead to significant health harms. Psychological distress occurs when stress experiences are either chronic, severe, or both [4]. Addressing the psychological impacts of COVID-19 is a major undertaking as nations navigate new stages of the global pandemic [4].

Neighborhoods, both their physical and social environments, may have acted either as sources of stress or buffer against psychological distress throughout the pandemic. Stay-at-home orders were frequently enacted to avoid virus transmission at places of education and employment. Thus, homes and neighborhoods became predominant exposure environments for many people during the pandemic. Neighborhood conditions resulting from structural racism often lack health-supporting resources such as clean air, healthy food, quality housing, parks, and services [5], and may exacerbate negative outcomes associated with a global pandemic such as COVID-19. Divested neighborhoods can affect health behaviors [6] and social systems. For example, neighborhoods that lack walkability (pedestrian-friendly with nearby amenities), key amenities, and have high rates of violence may reduce an individuals’ abilities to leave their homes, while also potentially increasing psychological distress. Some neighborhood conditions such as violent crime rates and off-premise liquor store densities can reduce collective efficacy, which refers to the degree to which neighbors work together to exert social control and achieve common goals [7] and other markers of social capital [8,9], thereby potentially impacting mental health.

On the other hand, neighborhood factors may act as buffers of distress associated with COVID-19. A growing number of population-based longitudinal studies have established lower psychological distress associated with improved exposure and access to parks and greenspaces [10,11,12]. Parks and other neighborhood amenities may also decrease distress given their impact on neighborhood attachment, trust, and a sense of belonging or connection [13,14]. These findings support hypotheses that are informed by theories of collective efficacy. For example, lower perceived collective efficacy was found to predict more frequent psychological distress following the 2005 hurricane season in Florida [15]. Neighborhood conditions that do not support social interactions may contribute to isolation.

The purpose of this study was to examine changes in psychological distress following the emergence of the COVID-19 pandemic, and the potential buffering or exacerbating role of neighborhood conditions on such changes.

## 2. Materials and Methods

### 2.1. Study Location and Design

This study took place in New Orleans, Louisiana, which has been especially hard-hit by the pandemic. In Louisiana, the COVID death rate for Black communities was more than double the rate compared to other racial and ethnic groups, and in New Orleans, Blacks account for more than 70% of COVID-related deaths, while making up roughly 59% of the total population [16,17]. The changes resulting from COVID-related mandates and phases of the city’s reopening have led to varying levels of economic, social, and health-related disruptions and adjustments [18].

This analysis was based on data collected as part of a cluster randomized trial called the Healthy Neighborhoods Project (HNP). HNP has been implemented in 23 neighborhoods in New Orleans to examine the impact of vacant land and property remediation on violence, health, and social outcomes. The study includes a residential cohort of approximately 400 participants surveyed over four waves, which began in January 2019, and is estimated to continue until 31 December 2023. Participants were recruited from 194 randomly-sampled trial clusters of 1/8 mile radius, and all residents from each cluster were invited to participate in the study. Individual participant sampling was initially random, but due to lower population numbers in some clusters, all residents were invited to participate, so the resulting sample is a convenience sample. The current analysis is based on a sub-sample of 244 respondents with baseline data collected prior to the pandemic (“pre-pandemic”; January 2019–March 2020), and wave two data collected following the stay-at-home orders (“during-pandemic”; 20 March 2020–19 April 2021) [19].

### 2.2. Data and Measures

#### 2.2.1. Data Collection

Trained interviewers collected survey data utilizing REDCap™ software (REDCap, Nashville, TN, USA) in person (5%) and over the phone (95%). Participants with different modes of data collection did not differ in any way according to sociodemographic factors, exposures, or outcomes of interest in the present study. Both pre-pandemic and during-pandemic surveys took approximately 45 min to complete, and respondents provided consent to participate prior to enrollment. The Tulane University Institutional Review Board approved the study. Informed consent was obtained from all subjects involved in the study.

#### 2.2.2. Outcome Measures

The primary dependent variable in the present study was psychological distress, measured by the validated and widely utilized Kessler 6 (K6) Psychological Distress Scale [20]. The scale measures the frequency of “non-specific psychological distress” [21] with six items, each ranging from zero for “none of the time” to four for “all of the time”. Questions focus on negative feelings/emotions and related ability to carry-out normal activities and care-seeking. The items are reduced into a summary score, with higher scores indicating greater psychological distress [20]. Scores of 5 and over indicate moderate or serious psychological distress, and 13 and over indicate serious psychological distress [21]. The index demonstrated internal reliability in the HNP sample (Cronbach’s alpha = 0.89).

We operationalized change in distress by the Kessler index difference score between pre-pandemic and during-pandemic. A difference greater than zero indicates an increase in self-reported distress, and less than zero indicates a decrease in self-reported distress among the sampled households. We further operationalized change as an increase in distress versus no change or decrease since the start of the pandemic, which allowed us to identify any self-reported changes, and possible psychometric associations between the two time periods [22].

#### 2.2.3. Exposures

We examined the impact of several neighborhood conditions as potential buffers or exacerbators of psychological distress during the pandemic. These included perceived collective efficacy, Walk Score^®^ and ParkScore, total crime rate, rate of homicides and assaults, and the neighborhood alcohol environment including both on- and offsite outlet density. We measured all exposures at the census block group level, which we defined as our ‘neighborhood’ unit. Orleans parish comprises 3471 census block groups and there was a total of 143 block groups in the sample, with an average of two people per block group (range = 1 to 5).

We examined a commonly employed marker of collective efficacy, perceptions of collective efficacy, developed by Sampson and colleagues [23]. The scale includes markers of social cohesion (sharing values with neighbors) as well as trust. The questions are measured on a 5-point Likert scale ranging from 1 (strongly disagree) to 5 (strongly agree). Overall collective efficacy is measured by totaling across items, with a higher score indicating a stronger perception of neighborhood level collective efficacy. The scale demonstrated high reliability in our sample (Cronbach’s alpha = 0.84). Scores were averaged to the block group level.

Walk Score^®^ provides a measure of neighborhood walkability [24]. It ranges from 0 to 100 (least to most walkable) and is calculated using information about the proximity of nearby land uses and facilities (educational, commercial, food-related, recreational, and entertainment). We used the ParkScore index as a measure of park access [25]. The ParkScore represents quality of urban park systems based on park acreage (median park size and park area as a percentage of city land area), park access (percent of the city population living within a 10-min walk to a park), and facilities and investment (measures of spending on parks and recreation per resident and a per capita average of recreation amenities). ParkScore can vary between 0 and 100, with higher scores describing park systems with higher quality. As with the Walk Score, the ParkScore Index was available at the block group level.

Crime data from 2019 was based on 911 calls reporting crime events and police reports from the New Orleans Police Department. Neighborhood total crime rate represented the sum of calls reporting assault, homicide, robbery, and crime by weapon per block group divided by the block group population for a rate per 1000 residents. Similarly, neighborhood homicide and assault rates represented the sum of calls reporting homicides and assaults in each block group divided by the population for a rate per 1000 residents.

Alcohol outlet density was assessed with geocoded alcohol outlet locations [26]. Numbers of active alcohol outlets by block group were tabulated for off-premise (e.g., liquor stores) and on-premise (e.g., bars) establishments. Alcohol outlet density for offsite and onsite establishments represented the total number of offsite and onsite outlets per block group divided by the block group population per 1000 residents.

#### 2.2.4. Covariates

We included covariates related to understanding the neighborhood factors and their potential impact on changes in self-reported psychological distress including relationship status (“married”, “living with partner”, “divorce/separated”, “widowed”, “single/never married”, and “other”), sex, age (years), education (“less than high school”, “high school graduate”, “some college”, “4-year college”, and “graduate or professional” levels), employment status (“full-time”, “part-time”, “unemployed”, “unable to work due to disability”, and “other”, which included individuals who were retired, full time homemakers, or in school or a training program), social support, and self-reported racial and ethnic identity variables, which could affect access to resources that may be relevant to health and mental health outcomes.

We measured during-pandemic social support utilizing the Brief 2-Way Social Support Scale [27], which includes receipt of emotional and instrumental support, with question six added from a previous version of the scale [28]. Respondents indicated whether it was (0), not at all true, to (5) always true for the following statements: “there is at least one person that I can share most things with”, “when I am feeling down there is someone I can lean on”, “there is someone in my life I can get emotional support from”, “if stranded somewhere, there is someone who would get me”, “I have someone to help me if I am physically unwell”, “there is someone who would give me financial assistance”, and “there is someone who can help me fulfill my responsibilities when I am unable”. We summed responses to create a social support index, which demonstrated high reliability in this sample (Cronbach’s alpha = 0.91).

### 2.3. Statistical Analysis

We performed analyses with SAS version 9.4 (SAS Institute, Inc., Cary, NC, USA). We first compared Census tract-level demographic characteristics of the study sample and the city of New Orleans. We then conducted statistical tests (McNemar’s test, paired *t*-tests) for pre-during COVID-19 differences in psychological distress and neighborhood exposure variables. In addition, we ran bivariate regression models to examine unadjusted associations between change in distress and neighborhood exposures.

We then ran crude and adjusted modified linear regression models with generalized estimating equations, robust variance estimation, and compound symmetry working correlation structure, clustering by census block group to model the relation between neighborhood exposures and change in distress over time (difference score). We ran models for each neighborhood exposure (independently), and modeled the control variables as fixed effects. We considered variables that created 10% or greater difference between the unadjusted and adjusted effects as confounders and controlled for them in the final model. Multicollinearity was assessed by examining correlations between covariates and variance inflation factors (VIFs) and did not include any measure that had a value of 7.0 or greater for VIF. We tested model fit using goodness of fit statistics including Akaike’s information criterion (AIC), the maximum likelihood ratio, and quasi-likelihood information criteria (QIC). We calculated the intraclass correlation (ICC) to determine the amount of clustering at the neighborhood-level using an unconditional means model. To account for potential structural confounding or endogeneity, with non-random choice of residence [29], we calculated the propensity score (PS) for differences in the likelihood of living in areas with high or low exposure to the neighborhood condition of interest. Results revealed significant overlap between those with high and low propensity for exposure, suggesting comparability across the sample and no need to match based on PS. Finally, we assessed effect modification by social support and sex by including interaction terms in the fully-adjusted regression model. Given the reduction in power for interaction terms, *p* < 0.20 was used to determine statistical significance. We used stratification to examine interactions that were statistically significant at *p* < 0.20 [30].

## 3. Results

**Figure 1:**
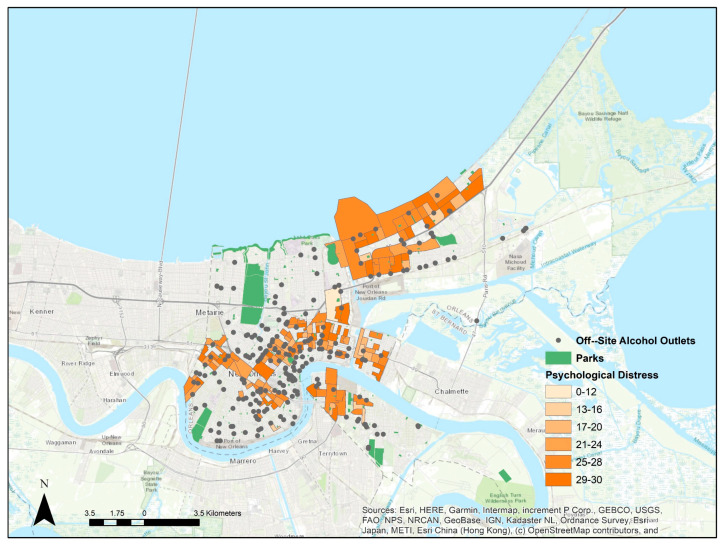
Map of the study area showing psychological distress scores by block group, and locations of parks and off–site alcohol outlets. Only block groups with study participants are shown.

The study area including block-group level baseline distress and locations of alcohol outlets and parks, is shown in Figure 1. Table 1 shows the census tract-level demographic comparison of the city of New Orleans and the study sample. Study participants live in neighborhoods that have higher unemployment, lower education levels, and higher percent Black residents than the city overall.

The demographic characteristics of study participants and neighborhood characteristics at baseline, stratified by changes in psychological distress, are presented in Table 2. Nearly half (47.5%) of participants experienced an increase in distress. The average age of participants was 52 years (range 22–94). Approximately 70% were female, 80% self-identified as Black, and 4% Hispanic. Forty percent of participants were single, 26% were married or living with a partner, 10% widowed, 11% divorced or separated, and 14% other. Forty percent had full-time employment and unemployment was slightly higher among participants whose distress increased compared to decreased or did not change (10% vs. 5%, respectively). While not shown, the proportion of those unemployed increased from approximately 7% to 28%. The mean distress score was 0.81 with a standard deviation of 4.68 and a range of −14 to 13 (not shown in table). Participants who experienced an increase in distress were significantly more likely to have completed high-school and have higher education compared to those who reported a decrease or no change in distress (*p* < 0.05).

With the exception of perceived neighborhood collective efficacy and onsite alcohol density, participants who experienced an increase in distress had greater average levels of all neighborhood exposures. The average perceived neighborhood collective efficacy score was 28 (range 8–40), with scores slightly lower for participants who experienced an increase in distress (*p* < 0.05). Average neighborhood walk and park scores were 55 (range 1–96) and 66 (range 0–100). Neighborhood onsite alcohol outlet density was 1.5 per 1000 population (range 0–18) and 0.89 per 1000 population (range 0–10) for offsite alcohol outlet density. Participants who experienced an increase in distress lived in neighborhoods with higher total crime, assault, and homicide rates compared to those who experienced a decrease or no change. Results of an unconditional means model suggested moderately high clustering of psychological distress by neighborhood block group, with approximately 6% of the variance in distress score explained at the neighborhood level (ICC = 0.63).

The results of crude and adjusted models for pre-post COVID distress difference score by neighborhood exposure variables are presented in Table 3. In unadjusted crude models, perceived neighborhood collective efficacy was significantly and inversely associated with distress score, with a 0.37 unit decrease in distress score for each increase in perceived neighborhood collective efficacy score (*b* = −0.37, *p* < 0.01).

However, after controlling for age, sex, relationship status, education, employment, and social support, we observed several additional associations, as shown in Table 3. The relationship with perceived neighborhood collective efficacy, while weaker, remained. We also observed significant positive associations between change in psychological distress and walk score (*b* = 0.05, *p* < 0.01), park score (*b* = −0.03, *p* < 0.01), assault rate (*b* = 0.14, *p* < 0.01), and offsite alcohol outlet density (*b* = 0.89, *p* < 0.01) and an inverse association with homicide rate (*b* = −1.20, *p* < 0.01).

Effect moderation by sex and social support was also observed for many neighborhood exposures. Table 4 depicts the association in each stratum for exposures where interaction terms were statistically significant. For sex, once stratified, we found that neighborhood walk score was significantly associated with a lower level of distress among men (*b* = −0.05, *p* < 0.01), but confidence intervals between men and women overlapped for most exposures. Greater levels of perceived collective efficacy, walk score, homicide rate, and onsite alcohol density were associated with increases in distress for participants with high levels of social support. However, neighborhood assault rate and offsite alcohol density were inversely associated with distress change among those with high levels of social support. Among participants with lower levels of social support, neighborhood walk score and assault rate were significantly and positively associated with distress score. However, only the associations between homicide and assault rates and distress did not have overlapping confidence intervals.

## 4. Discussion

This study is one of the first to examine the association between neighborhood buffers and risks on psychological distress pre-post COVID-19 in a primarily Black population [31]. While people of color who have experienced disproportionately higher cases and deaths from COVID-19 [32] are also at higher risk for psychological distress [33], neighborhood characteristics may buffer this risk. After adjusting for potential confounders, we found that higher perceived collective efficacy, homicide rate, and park score were associated with a decrease in distress. Greater collective efficacy has been found to predict lower psychological distress in the event of prior disasters [15]. Greater access to parks and greenspaces, outside of the COVID context, has been associated with lower psychological distress [10,11,12]. Parks have been shown to be key neighborhood amenities under quarantine conditions for mental health. While Astell-Burt et al. [11] reported physical activity to be a potential mechanism of this association, we found that higher Walk Score was associated with a decrease in distress only among men and those reporting high levels of social support. It could be that walkable neighborhoods, typically characterized by higher-density mixed land use, triggered higher distress associated with fear of the virus and closure of nearby amenities. Walkable areas may also have less green space, which may reduce distress [34]. Walkability may also have triggered distress related to fear among women, who may experience and perceive neighborhood environments differently than men [35].

While a higher assault rate was associated with increase in distress, higher homicide rate was associated with a decrease in distress. Prior research has found that an increase in neighborhood crime is associated with an increase in distress [36]. Other research has found that while higher perceived crime often predicts higher distress [37,38], exposure to more reported violent crime is not always associated with higher distress [39]. These findings could indicate higher awareness of local homicides compared to assaults and that residents have become desensitized to homicides. Desensitization to violence, as a form of habituation, involves learning to decrease or dimmish one’s response after repeated exposure [40]. Homicides could also trigger subsequent strengthening of support and protection for loved ones [41]. Previous research has demonstrated that social mechanisms have also helped Black Americans build resilience in the face of disproportionate harmful exposures to structural racism [42]. These social mechanisms include group coping, often tied to religious institutions and general social support [43].

We also found that higher offsite alcohol density was associated with an increase in distress score, especially among individuals with high levels of social support. High alcohol outlet density has been associated with both an increase in alcohol consumption and an elevated risk of mental health-related hospital admissions [44], outside of the COVID context. COVID-19 and quarantine conditions triggered an increase in alcohol abuse [45], and higher outlet density may have supported this increase and any alcohol-related violence or mental health issues that often result [46,47]. In some locations, alcohol outlets closed during the quarantine, which could trigger withdrawal among those suffering from alcohol use disorders [48] and potential increases in distress.

Social support can play a role in the neighborhoods’ influence on mental health and in this sample, it was an important effect modifier. Among participants with high levels of social support, distress was greater for those with higher perceived collective efficacy, walk score, homicide rate, and onsite alcohol density. Among those with high levels of social support, assault rate and off-premise alcohol density were also associated with decreasing distress. Among participants with lower levels of social support, neighborhood walk score and assault rate were significantly associated with increasing distress. One explanation may be that for those with higher levels of support, perceived collective efficacy and walkable surroundings may be more connected and therefore more distressed by COVID events given a wider social network. For those with lower support, perhaps closure of local amenities triggered distress. Related work has found that the relationships between various measures of community social connections and COVID-19 cases and deaths is not straight-forward [49].

### Limitations

This study is not without limitations. Our study sample came from a relatively low-income and high-violence areas in a Southern U.S. city, and our findings may not be generalizable outside of this sample and the study context. We used a convenience sample, which presents potential selection bias and structural confounding caused by neighborhood selection not being a random event. However, propensity scored analysis showed substantial overlap between participants in the exposed and non-exposed groups, suggesting comparability of participants and limited structural confounding.

Our outcome measures are self-reported survey responses, and therefore information recall and social desirability may affect responses. Our exposure measures such as ParkScore and Walk Score represent calculated neighborhood characteristics that may not accurately reflect access or exposure to parks or greenspace, or the walkability of a block or neighborhood. In addition, reported crime events from the police department may be subject to reporting bias—in some neighborhoods, there may be barriers to the reporting of crime and violence due to fear of retribution. Finally, the number of residents per block group was small for some areas, however, this may not have been a significant issue given the size and number of groups [50].

## 5. Conclusions

Neighborhoods play a central role in mental and physical health, particularly during disasters and crises such as the COVID-19 pandemic. This may be particularly true for those who have experienced disproportionately high cases and deaths from COVID-19. While higher densities of offsite alcohol outlets, assault rates, and walkable streets in neighborhoods were associated with an increase in distress, neighborhood parks as well as collective efficacy were associated with reduced distress among our participants. Results also point to the importance of considering heterogenous effects of the neighborhood environment on distress, with potentially differential impacts by sex and level of social support in someone’s life. The relationships between both social and physical characteristics of neighborhoods and interpersonal and individual factors that may be linked to distress are complicated and should be explored in greater detail.

## Figures and Tables

**Table 1:** Census tract-level demographic comparison of the city of New Orleans and the study sample.

	Mean/%	SD	Range
Census Tract (Orleans)			
Age	43.60	10.11	(20–89.27)
% Unemployed	9.03	5.8	(0–35.51)
% Less than high school education	15.09	11.02	(0–54.90)
% Black	57.12	33.15	(0–99.13)
Census Tracts in Neighborhoods of Sample			
Age	42.36	8.44	(24.56–83.42)
% Unemployed	12.60	5.47	(0–35.52)
% Less than high school education	21.57	7.79	(2.20–54.90)
% Black	81.63	18.11	(9.37–99.12)

**Table 2:** Individual and neighborhood socio-demographic characteristics of study participants stratified by psychological distress before and during COVID-19.

	Increase in Psychological Distress (*n* = 116)	No Change or Decrease in before Psychological Distress (*n* = 128)	Total (*n* = 244)
	Mean (Range)/% ^1^	Mean (Range)/%	Mean (Range)/%
Average age (years)	50.91 (22–83)	53.38 (22–94)	52.20 (22–94)
Sex: Female	69.83%	69.53%	69.67%
Sex: Male	30.17%	30.47%	30.33%
Self-reported racial identity:			
Asian	0.00%	0.86%	0.41%
Black	80.20%	77.30%	78.96%
White	12.93%	17.97%	15.57%
Multi-racial	6.03%	4.69%	5.33%
Self-reported Hispanic ethnicity	3.13%	4.31%	3.69%
Relationship status:			
Married/living with a partner	26.72%	24.22%	25.41%
Divorced/Separated	9.48%	12.50%	11.07%
Widowed	10.34%	10.16%	10.25%
Single	38.69%	39.84%	39.34%
Other	14.65%	13.29%	13.94%
Employment status:			
Full-time	37.93%	39.06%	38.52%
Part-time	11.21%	14.84%	13.11%
Unemployed	9.48%	4.69%	6.97%
Unable to work due to disability	14.66%	17.19%	15.98%
Other	26.72%	24.22%	25.41%
Education ^2^: Less than high school	5.31%	13.71%	9.70%
Education ^2^: High school graduate	32.74%	26.61%	29.54%
Education ^2^: Some college	36.28%	24.19%	29.96%
Education ^2^: 4-year college	17.70%	22.58%	20.25%
Graduate or professional school	7.96%	12.90%	10.55%
Average reported social support	30.72 (14–35)	31.18 (0–35)	30.96 (0–35)
Average perceived neighborhood collective efficacy ^2^	26.10 (8–40)	28.94 (13–40)	27.57 (8–40)
Average neighborhood walk score	55.52 (1–95)	53.26 (1–96)	54.33 (1–96)
Average neighborhood park score	66.22 (0–100)	65.92 (0–100)	66.07 (0–100)
Average neighborhood total crime rate ^3^	99.21 (8.04–347.64)	98.93 (21.38–347.64)	99.07 (8.04–347.64)
Average neighborhood homicide rate ^3^	0.82 (0–7.98)	0.64 (0–5.62)	0.72 (0–7.98)
Average neighborhood assault rate ^3^	7.19 (0–37.40)	6.79 (0–37.39)	6.98 (0–37.40)
Average neighborhood onsite alcohol outlet density ^3^	1.40 (0–14.45)	1.67 (0–17.83)	1.54 (0–17.83)
Average neighborhood offsite alcohol outlet density ^3^	1.01 (0–9.93)	0.77 (0–6.43)	0.89 (0–9.93)

^1^ Mean/% of any variable based on <10% missing. ^2^ Statistically significant difference at *p* ≤ 0.05 in *t*-test or chi-square. ^3^ Per 1000 population.

**Table 3:** Impact of neighborhood conditions on psychologic distress before and during COVID-19: Results of crude and adjusted general estimating equation (GEE) models ^1^.

	Crude Models			Adjusted Models ^2^		
	*b*	95% CI	*p*-Value	*b*	95% CI	*p*-Value
Perceived neighborhood collective efficacy	−0.37	−0.61, −0.13	<0.01	−0.23	−0.35, −0.09	<0.01
Neighborhood walk score	0.70	−0.22, 1.62	0.13	0.05	0.02, 0.07	<0.01
Neighborhood park score	−0.31	−0.81, 0.18	0.22	−0.03	−0.05, −0.01	<0.01
Total neighborhood crime rate	0.08	−0.10, 0.26	0.40	0.01	−0.00, 0.02	0.10
Neighborhood homicide rate	2.62	−5.59, 10.82	0.53	−1.20	−1.80, −0.60	<0.01
Neighborhood assault rate	0.63	−1.38, 2.64	0.54	0.14	0.03, 0.24	<0.01
Neighborhood onsite alcohol density	3.31	−0.52, 7.14	0.09	0.19	−0.11, 0.48	0.21
Neighborhood offsite alcohol density	1.31	−0.12, 2.75	0.07	0.89	0.52, 1.23	<0.01

^1^ Modeling difference score in K6. Greater difference score indicates an increase in psychological distress following the start of the COVID pandemic. ^2^ Controlling for age, sex, relationship status, education, employment, and social support.

**Table 4:** Impact of neighborhood conditions on psychologic distress before and during COVID-19: Results of adjusted ^1^ general estimating equation (GEE) stratified by sex and social support.

	*b* (95% CI) ^2^			
	Female (*n* = 170)	Male (*n* = 74)	High Social Support (*n* = 120)	Lower Social Support (*n* = 119)
Perceived collective efficacy	---	---	0.06 ** (0.03, 0.09)	−0.08 (−0.27, 0.12)
Neighborhood walk score	0.02 (−0.01, 0.05)	−0.05 * (−0.11, 0.00)	0.03 ** (0.02, 0.04)	0.05 ** (0.01, 0.09)
Neighborhood total crime rate	---	---	−0.01 (−0.03, 0.01)	−0.02 (−0.01, 0.01)
Neighborhood homicide rate	0.01 (−0.46, 0.48)	−0.28 (−1.00, 0.44)	1.85 ** (0.36, 3.34)	−0.16 (−0.72, 0.41)
Neighborhood assault rate	---	---	−0.15 ** (−0.19, −0.12)	0.23 ** (0.09, 0.37)
Neighborhood onsite alcohol density	---	---	0.43 ** (0.34, 0.51)	0.02 (−0.41, 0.45)
Neighborhood offsite alcohol density	0.11 (−0.35, 0.57)	0.26 (−0.13, 0.65)	−0.82 ** (−1.07, −0.57)	0.09 (−0.28, 0.47)

^1^ Controlling for age, relationship status, education, employment. ^2^ *p*-value: * <0.1; ** <0.05.

## Data Availability

Parent study is not yet complete and per NIH sharing plan, not yet available.
